# Analysis of the Immunoexpression of Opioid Receptors and their Correlation with Markers of Angiogenesis, Cell Proliferation and Apoptosis in Breast Cancer

**DOI:** 10.31557/APJCP.2021.22.2.633

**Published:** 2021-02

**Authors:** Alceu Machado de Sousa, Thinali Sousa Dantas, Paulo Goberlânio de Barros Silva, Conceição da Silva Martins, Gildenio Estevam Freire, Howard Lopes Ribeiro Junior, Gerly Anne de Castro Brito, Karuza Maria Alves Pereira, Renata Ferreira de Carvalho Leitão

**Affiliations:** 1 *Department of Morphology, Faculty of Medicine, Federal University of Ceará, Fortaleza, Ceará, Brazil. *; 2 *Division of Oral Pathology, Faculty of Pharmacy, Dentistry and Nursing, Federal University of Ceará, Fortaleza, Ceará, Brazil. *

**Keywords:** Luminal A, luminal B- mu-opioide receptor, kappa, opioide receptor, prognosis

## Abstract

**Objective:**

Breast cancer is a disease of great concern. The prognosis of this tumor is related to its staging. Opioids are widely used to minimize pain in oncology clinics; however, the relationship between the administration of opioids and their effects on tumor cells has yet to be elucidated. Therefore, this study aimed to evaluate the immunoexpression of mu- (μ) and kappa- (κ) opioid receptors and their correlation with markers of angiogenesis, cell proliferation, and apoptosis in biopsies of breast tumors.

**Methods:**

Demographic data, tumor characteristics, opioid use, and prognostic factors were collected from medical records. After the selection of the excisional biopsies, immunohistochemistry was performed for μ- and κ-opioid receptors, vascular endothelial growth factor (VEGF), Ki-67, and TUNEL.

**Results:**

A significant predominance of Ki-67 and μ-opioid receptor immunoexpression in the lymph nodes was observed in patients administered opioid medications. The luminal A subtype showed higher apoptosis levels (TUNEL) compared to the luminal B subtype. Patients with T4 tumor who had recurrence demonstrated a reduced expression of κ-opioid receptors at the lymph node location. Correlation analyses between the μ and κ opioid markers, VEGF, Ki-67, and TUNEL showed that these findings are likely involved in the same mechanisms the cancer of T4 stage breast cancer.

**Conclusion:**

The κ-opioid receptor has a lower immunoexpression in nodal tumor metastasis with recurrence, whereas the μ-opioid receptor is directly related to expression of TUNEL-positive cells in tumors and indirectly to Ki-67 in nodal metastasis. Neither of the two receptors was expressed in the primary tumor or nodal metastasis in relation to VEGF.

## Introduction

Breast cancer is the most prevalent cancer in women worldwide (Bray et al., 2018). The number of new breast cancer cases in Brazil has been estimated at 59,700 between 2018 and 2019, with an approximate risk of 56.33 cases per 100,000 women, according to the Brazilian National Cancer Institute (INCA, 2020). These estimates indicate the need to develop new methods of prevention, diagnosis, and treatment for breast cancer. Some associated factors have already been established to predict the aggressiveness of the disease. In some cases, the treatment protocol to be adopted in daily clinical practice depends on factors, such as the number of axillary lymph nodes involved, histological type and grade, angiolymphatic and perineural invasion, as well as the expression of some hormone receptors (Estrogen Receptor (ER), Progesterone Receptor (PR)), and c-erbB2/HER2. Moreover, tumor size also plays a significant role in the recovery process, considering that patients with large tumors have worse prognosis (Sotiriou et al., 2006).

In addition to compromising patient prognosis, large tumors often lead to more suffering caused by painful symptoms. In the United States, 30% to 40% of newly diagnosed cancer patients and 90% of advanced cancer patients report moderate and severe pain, which progresses in relation to tumor size, degree of metastasis, type, and location of the tumor (Koodie et al., 2010). Therefore, opioid drugs are prescribed for many cases, as they are the most effective drugs currently available for pain control in oncology (Gach et al, 2011).

The pharmacology and analgesic function of opioids in the central nervous system are well documented (Al-Hasani and Bruchas, 2011). However, little is known about their effects on tumor cells. For this reason, interest in investigating the possible effects of opioids, especially morphine, on tumor progression has increased in the last decade (Gach et al., 2011). However, the results obtained so far are conflicting. Discrepancies in the results are likely due to the different cell lines investigated in each study, the various concentrations of morphine tested, or the mode of administration.

Literature shows that morphine influences the inflammatory response in the tumor microenvironment, interacting with the common signaling pathways through anti-inflammatory and pro-inflammatory responses. It has been suggested that Mu- (μ) and Kappa- (κ) opioid receptors have opposite roles, the former being related to the induction of pro-inflammatory activity, and the latter, to anti-inflammatory activity (Finley et al., 2008).

Therefore, the aim of this study was to evaluate the expression of μ- and κ- opioid receptors correlating prognostic factors, angiogenesis marker, cell proliferation, and death of Luminal A and Luminal B breast cancer cells.

## Materials and Methods

This study was approved by the Human Research Ethics Committee of the Haroldo Juaçaba Hospital/Cancer Institute of Ceará (HJH/CIC), under protocol number 1,618,068.

This is an observational, quantitative, retrospective, and cross-sectional study which was conducted between January 2011 and December 2016 at HJH/CIC. Excisional biopsy specimens from 39 patients with Luminal A (n=11) and Luminal B (n=28) breast cancer, T4 tumor size, with and without metastatic process were selected. Socio-demographic data, such as age, sex, educational level, race, healthcare coverage (public or private) as well as clinical-pathological aspects comprising histological characteristics, tumor staging, tumor immunohistochemical profile, and treatment protocol were collected from the patients’ medical records. Patients who were male, provided with insufficient material for analysis, and had incomplete medical records were excluded from the study.

After patient selection, an experienced pathologist independently analyzed the excisional biopsies and chose fragments of metastatic lymph nodes and peritumoral region to produce tissue microarray (TMA) blocks. 

For the TMA technique, a hollow needle (Quick-Ray UNITMA^®^) was used to remove small tissue fragments of 0.6 mm in diameter. These fragments were then inserted into a paraffin receptor block, in a precisely spread matrix pattern. Each microarray block was composed of 60 tissue fragments. The receptor block was then divided into 3 μm thick sequential sections that were deposited on glass slides and stained with conventional hematoxylin-eosin (HE) staining, followed by immunohistochemical analysis with the antibodies for anti-mu opioid receptor (1: 100) Abcam®, anti-kappa opioid receptor (1: 200) Abcam^®^, anti-vascular endothelial growth factor (VEGF) (1: 200) Abcam^®^, and anti-Ki67 (1: 100) Abcam^®^.

The immunohistochemical reaction was performed by means of the streptavidin-biotin-peroxidase technique adapted from HSU et al., (1981). After dewaxing and rehydration, antigen retriev,al was performed using citrate buffer (pH 6.0) in a 97°C water bath for 45 minutes, blocking the endogenous peroxidase with hydrogen peroxide, followed by washing with phosphate buffered saline (PBS) solution and overnight incubation with the primary antibodies.

After incubation with the primary antibody, the slides were washed with PBS and incubated with Envision Flex/HRP (polymer) DAKO system for 30 minutes. Subsequently, the slides were washed with PBS.

The slides were stained with 3,3’-Diaminobenzidine-peroxide (DAB) (Dako®), counterstained with Harris Hematoxylin (10 “) and mounted in Entellan®️

Controls were considered positive from the human placenta to the κ-opioid receptor and the human brain to the μ-opioid receptor.

*TUNEL assay*

Tissue sections (3.0 μm) were digested for antigen retrieval with Proteinase K (1: 250) for 15 minutes and peroxidase activity was blocked with a 3% H2O2 solution diluted in PBS for 5 minutes. After peroxidase blocking, the slides were incubated for 10 seconds with a ready-to-use buffer and Tdt (1: 2.5) for 90 minutes at room temperature.

The stop solution was diluted in wash buffer (1:15) for 10 minutes. After washing, the anti-digoxigenin (ready-to-use) conjugate was added. The color development system used was DAB and the counterstaining was performed with methyl green for 10 minutes followed by dehydration, diaphanization, and assembly with coverslips.

For microscopic evaluation, five fields per histological section were photographed at 400× magnification using a light microscope coupled with a camera (Leica DM 2000®). The samples were then submitted to categorical and quantitative/qualitative evaluation (Zhang et al., 2013). Brownish pigmentation of the nucleus, cytoplasm, or both was considered as positive staining.

In the qualitative evaluation, the following scores were determined for the immunostaining intensity: 0 = no immunomarking; 1 = light immunomarking; 2 = moderate immunomarking; and 3 = intense immunomarking. For the quantitative evaluation, images were exported to the ImageJ® software and, through the cell counter command, the percentage of stained and unstained tumor cells in representative areas of the tumor, peritumor, and lymph node metastasis were determined. This percentage was categorized from 1 to 4: 1, 0-25% positive cells; 2, 26-50% positive cells; 3, 26-75% positive cells; and 4, 76% -100% positive cells.

For statistical purposes, histoscores were determined by multiplying the intensity scores and the percentage of stained cells, resulting in the following score: 0-4, negative or light staining; 5-8, moderate staining; and 9-12, intense staining. The markers evaluated by percentage were Ki-67 and TUNEL.

*Statistical analysis*

Quantitative data were subjected to the Kolmogorov-Smirnov normality test and analyzed by the Mann-Whitney test and Spearman’s non-linear correlation. Categorical data were expressed as absolute and percentage frequency values and were analyzed using Fisher’s Exact or Chi-square test.

The level of statistical significance was p <0.05 (2-sided test) and all analyses were performed using SPSS software for Windows version 20.0 (SPSS Inc., Chicago, IL, USA). 

## Results

*Sample characterization and risk factors for recurrence*

A total of 49 patients were evaluated, with the majority being of mixed-race (n = 31, 79.5%), ≤60 years of age (n = 20, 51.3%), having some type of affective bond (n=26, 66,7%), originally from the countryside (n=21, 53.8%), with complete primary education or lower educational qualification (n=22, 56.4%). Most individuals were publicly insured (n = 35, 92.1%), underwent surgery combined with radiotherapy (RT) and chemotherapy (CT) (n=31, 79.5%), exhibited nodal metastasis (n = 32, 80.1%), and had no signs of distant metastasis. The tumor phenotype of most cases was ER+ (n=37, 97.4%), followed by RP (n=34, 87.2%), and c-erbB2/HER2 (26.3%). The luminal B subtype was more prevalent (n = 28, 73.7%) than the luminal A subtype (n = 10, 26.3%) with recurrence in seven patients (17.9%). Two patients (5.1%) died during the study period. There was no association between these factors and recurrence (Table 1).

*Immunohistochemical profile*

**Figure 1:**
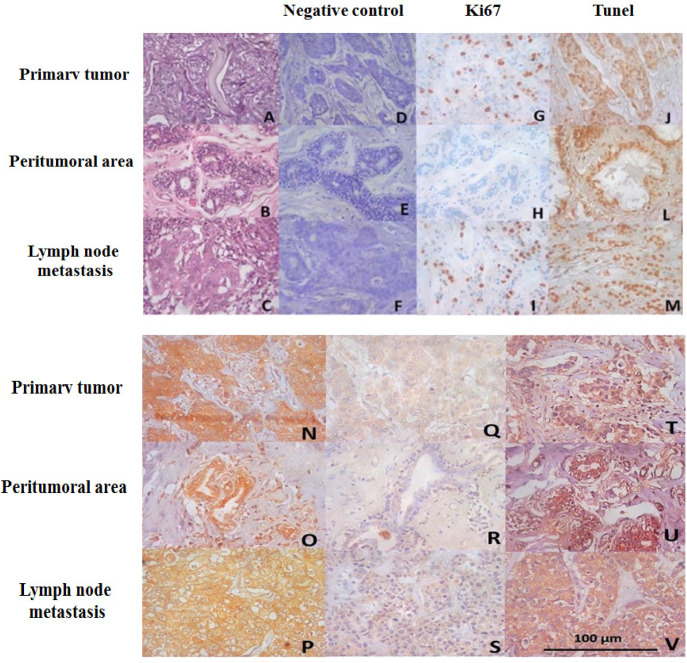
Photomicrograph of Primary Tumor HE (A) peritumoral area HE (B) and lymph node metastasis HE (C), negative control HE (D, E, F), and immunohistochemical profile for Ki67 (G, H and I), TUNEL assay (J, L and M), opiate kappa receptor lymph node (N, O and P), Mu opioid (Q, R, S) and VEGF (T,U, V). 400X magnification

Most tumors and nodal metastases were positive for Ki-67, demonstrating a staining percentage between 1-25%, whereas the peritumor region exhibited no immunostaining. The luminal B subtype showed 42.9% of cases with 25-50% of Ki-67 immunostaining; however, there was no significant difference between the luminal A and B subtypes (p = 1.000). Lymph node metastases showed a high prevalence of 1-25% Ki-67 immunostaining in both luminal A and luminal B subtypes. There were no significant differences between these two phenotypes (p = 0.484) (Figure 1).

TUNEL assay revealed a higher prevalence of scores (76-100%) in the tumor, perilesional area, and nodal metastasis. In the peritumoral region, the luminal B subtype showed greater expression of TUNEL than the luminal A variant (p = 0.007), but primary tumor (p = 0.472) and lymph node metastasis (p = 0.174) did not differ between luminal A and luminal B phenotypes (Table 2; Figure 1).

Immunoexpression of κ-opioid receptors was greater than µ-opioid receptors in all tissues, and there was no statistical difference in the immunoexpression of the phenotypes in luminal A and luminal B. (Table 3). The immunostaining for opioid receptors did not differ between recurrent or non-recurrent tumors and kappa-opioid receptors showed low immunoexpression in nodal metastasis of tumors with recurrence compared with tumors without recurrence (p = 0.033) (Table 4) (Figure 1). 

In the tumors, k-opioid was directly correlated with µ-opioid (p = 0.001) and TUNEL (p = 0.028); however, Ki-67 was inversely correlated with VEGF (p = 0.018). In the control tissues, µ-opioid and VEGF were directly correlated (p = 0.044), whereas the k-opioid was directly correlated with µ-opioid (p = 0.001) and inversely correlated with Ki-67 in the lymph node metastasis. Additionally, in lymph node metastasis, TUNEL-positive cells and VEGF were directly correlated (p = 0.047) (Table 6).

*VEGF immunostaining*

High VEGF scores were shown in both luminal A and luminal B phenotypes. There were no differences between these two subtypes in the tumors (p = 0.348), peritumoral area (p = 0.971), or lymph node metastasis (p = 0.830) (Table 4).

Despite VEGF immunostaining in primary tumors (p = 0.653) or peritumoral area (p = 0.574) did not show differences between recurrent and non-recurrent tumors; however, recurrence was inversely associated with VEGF immunostaining (p = 0.033) (Table 4).

**Table 1:** Descriptive Characterization of Patients' Clinical and Socio-Demographic Variables. in Relation to the Presence or Absence of Disease Recurrence

			Recurrence(n/%)		
	n	%	Without recurrence	With recurrence	p
Race					
Mixed/brown	31	79.5	26 (81.3)	5 (71.4)	0.617
White	8	20.5	6 (18.8)	2 (28.6)	
Age					
≤60 years	20	51.3	17 (53.1)	3 (42.9)	0.695
≥60 years	19	48.7	15 (46.9)	4 (57.1)	
Marital status					
Unmarried	18	46.6	9 (28.1)	4 (57.1)	0.194
Married	21	53.8	23 (71.9)	3 (42.9)	
Origin					
Metropolitan region	18	46.2	15 (46.9)	3 (42.9)	1
Countryside	21	53.8	17 (53.1)	4 (57.1)	
Educational level					
Primary	22	56.4	19 (59.4)	3 (42.9)	0.677
Higher education	17	53.8	13 (40.6)	4 (57.1)	
Health care coverage					
SUS	35	92.1	30 (93.8)	5 (83.3)	0.412
Private health insurance	3	7.9	2 (6.3)	1 (16.7)	
Treatment					
Surgery + RT	1	2.6	1 (3.1)	0 (0.0)	0.333
Surgery + CT	7	17.9	7 (21.9)	0 (0.0)	
Surgery + RT + CT	31	79.5	24 (75.0)	7 (100.0)	
N					
N0	7	17.9	6 (18.8)	1 (14.3)	0.886
N1	12	30.8	9 (28.1)	3 (42.9)	
N2	8	20.5	7 (21.9)	1 (14.3)	
N3	12	30.8	10 (31.3)	2 (28.6)	
M*					
MX	36	94.7	30(96.8)	6 (85.7)	0.339
M1	2	5.3	1 (3.2)	1 (14.3)	
ki 67*					
≤25%	17	48.6	14 (48.3)	3 (50.0)	1
≥ 25%	18	51.4	15 (51.7)	3 (50.0)	
Immunochemistry profile					-
RE +	37	97.4	-	-	
RP +	34	87.2	-	-	
HER2 +	10	26.3	-	-	
Phenotype*					
Luminal A	10	26.3	9 (29.0)	1 (14.3)	0.65
Luminal B	28	73.7	22 (71.0)	6 (85.7)	
Disease-free survival					
Without recurrence	32	82.1	-	-	-
With recurrence	7	17.9	-	-	
Survival					
Alive	37	94.9	-	-	-
Dead	2	5.1	-	-	

Pearson's chi-square test or Fisher's exact test, p<0,05. RT, radiotherapy; CT, chemotherapy

**Table 2:** Immunomarking for the Profile of Tumor Proliferation (ki67) and Cell Death (TUNEL) in Biopsy Samples from Patients with Breast AC Stratified According to the Molecular Subtype Luminal A and Luminal B

Molecular subtype						Logistic regression model			
Markes	Luminal A		Luminal B		p-Valor	P	OR	(CI 95%)	
Ki-67									
Primary tumor									
0%	3	30.00%	4	14.30%		-	-	-	-
1-25%	7	70.00%	13	46.40%		-	-	-	-
25-50%	0	0.00%	6	21.40%	0.25	-	-	-	-
51-75%	0	0.00%	2	7.10%		-	-	-	-
76-100%	0	0.00%	3	10.70%		-	-	-	-
Peritumoral area									
0%	0	0.00%	4	57.10%		-	-	-	-
1-25%	0	0.00%	3	42.90%		-	-	-	-
25-50%	0	0.00%	0	0.00%	1	-	-	-	-
51-75%	0	0.00%	0	0.00%		-	-	-	-
76-100%	0	0.00%	0	0.00%		-	-	-	-
Lymph node metastasis									
0%	0	0.00%	2	10.00%		-	-	-	-
1-25%	4	80.00%	10	50.00%		-	-	-	-
25-50%	0	0.00%	6	30.00%	0.484	-	-	-	-
51-75%	0	0.00%	1	5.00%		-	-	-	-
76-100%	1	20.00%	1	5.00%		-	-	-	-
TUNEL						-	-	-	-
Primary tumor									
0%	1	12.50%	1	3.40%		-	-	-	-
25-50%	1	12.50%	2	6.90%	0.472	-	-	-	-
51-75%	0	0.00%	4	13.80%		-	-	-	-
76-100%	6	75.00%	22	75.90%		-	-	-	-
Peritumoral area									
0%	1	12.50%	1	4.30%		-	-	-	-
25-50%	1	12.50%	0	0.00%	0.007*	-	-	-	-
51-75%	3	37.50%	1	4.30%		0.02	21	1.613	273.34
76-100%	3	37.50%	21	91.30%		-	-	-	-
Lymph node metastasis									
0%	1	11.10%	1	4.80%		-	-	-	-
1-25%	1	11.10%	0	0.00%	0.174	-	-	-	-
51-75%	0	0.00%	6	28.60%		-	-	-	-
76-100%	7	77.80%	14	66.70%		-	-	-	-

Pearson's chi-square test or Fisher's exact test, p<0,05

**Table 3.** Immunomarking for the Opioids Mu (µ), Kappa (k) and VEGF in Biopsies of Patients with Breast CA with Molecular Subtype Luminal A and Luminal B

Markers	Luminal A	Luminal B	p-Value
µ opioid (%)			
Primary tumor	3.30±4.40	3.29±3.36	0.897
Peritumoral area	0.83±1.60	1.30±2.67	1
Lymph node metastasis	3.86±3.58	4.33±3.51	0.836
κ opioid (histoescore)			
Primary tumor	10.67±2.65	11.08±1.75	0.987
Peritumoral area	9.50±5.00	11.42±1.38	0.721
Lymph node metastasis	9.83±2.56	10.40±2.87	0.796
VEGF (histoescore)			
Primary tumor	8.78±3.60	7.24±3.73	0.348
Peritumoral area	4.80±4.55	4.44±3.92	0.971
Lymph node metastasis	8.29±3.55	8.74±3.45	0.83

Mann-Whitney test; p<0.05 (mean ±SD)

**Table 4:** Immunomarking for the Opioids Mu (µ), Kappa (k) and VEGF in Relation to the Presence or Absence of Recurrence in Biopsy Samples from Patients with Breast Cancer

Markers	Without recurrence	With recurrence	p-Value
µ opioid (%)			
Primary tumor	3.75±3.80	1.50±1.97	0.245
Peritumoral area	1.43±2.38	0.00±0.00	0.233
Lymph node metastasis	4.77±3.73	2.60±3.44	0.286
VEGF (histoescore)			
Primary tumor	8.0±3.7	8.7±3.8	0.629
Peritumoral area	3.9±3.6	6.4±4.3	0.227
Lymph node metastasis	7.9±3.7	11.3±1.5	0.095
κ opioid (histoescore)			
Primary tumor	11.1±1.8	10.5±2.5	0.653
Peritumoral area	10.8±2.8	12.0±0.1	0.574
Lymph node metastasis	10.8±2.2	7.2±3.7	0.033*

Mann-Whitney test; p<0.05 (mean ±SD)

**Table 5:** Immunomarking for the Profile of Tumor Proliferation (Ki67) and Cell Death (TUNEL) in Relation to the Presence of Recurrence in Biopsy Samples from Patients with Breast Cancer

Markers	Recurrence (n/%)		
	Yes	No	p-Value
Ki67 (score)			
Primary tumor			
0%	7 (22.6)	0 (0.0)	
1-25%	15 (48.4)	5 (71.4)	
25-50%	4 (12.9)	2 (28.6)	0.38
51-75%	2 (6.5)	0 (0.0)	
76-100%	3 (9.7)	0 (0.0)	
Peritumoral area			
0%	2 (50.0)	2 (66.7)	
1-25%	2 (50.0)	1 (33.3)	
25-50%	-	-	1
51-75%	-	-	
76-100%	-	-	
Lymph node metastasis			
0%	2 (9.5)	0 (0.0)	
1-25%	10 (47.6)	4 (100.0)	
25-50%	6 (28.6)	0 (0.0)	0.547
51-75%	1 (4.8)	0 (0.0)	
76-100%	2 (9.5)	0 (0.0)	
TUNEL (escore)			
Primary Tumor			
0%	2 (6.7)	0 (0.0)	
25-50%	3 (10.0)	0 (0.0)	0.885
51-75%	3 (10.0)	1 (14.3)	
76-100%	22 (73.3)	6 (85.7)	
Peritumoral area			
0%	2 (7.7)	0 (0.0)	
25-50%	1 (3.8)	0 (0.0)	1
51-75%	3 (11.5)	1 (20.0)	
76-100%	20 (76.9)	4 (80.)	
Lymph node metastasis			
0%	2 (8.0)	0 (0.0)	
25-50%	1 (4.0)	0 (0.0)	1
51-75%	5 (20.0)	1 (20.0)	
76-100%	17 (68)	4 (80.0)	

Pearson's chi-square test or Fisher's exact test, p<0,05

**Table 6:** Correlation between Opioid Receptors and VEGF, ki-67 and TUNEL in Luminal A and Luminal B Breast Cancer

	µ opioid	VEGF	k opioid	Ki-67	TUNEL
Tumor					
µ opioid	-	p=0.115 (r=0.256)	*p=0.011 (r=0.408)	p=0.126 (r=-0.253)	*p=0.028 (r=0.361)
VEGF	-	-	p=0.211 (r=0.205)	*p=0.018 (r=-0.378)	p=0.627 (r=0.081)
k opioid	-	-	-	p=0.576 (r=-0.094)	p=0.965 (r=-0.008)
Ki-67	-	-	-	-	p=0.843 (r=-0.034)
TUNEL	-	-	-	-	-
Control					
µ opioid	-	*p=0.044 (r=-0.509)	p=0.555 (r=0.173)	p=0.374 (r=0.447)	p=0.579 (r=0.136)
VEGF	-	-	p=0.895 (r=-0.035)	p=0.237 (r=-0.514)	p=0.207 (r=0.280)
k opioid	-	-	-	p=1.000. (r=0.212.)	p=0.649 (r=0.119)
Ki-67	-	-	-	-	p=1.000 (r=0.343)
TUNEL	-	-	-	-	-
Lymph node metastasis					
µ opioid	-	p=0.406 (r=0.167)	*p=0.002 (r=0.568)	*p=0.047 (r=-0.400)	p=0.570 (r=-0.117)
VEGF	-	-	p=0.387 (r=0.170)	p=0.729 (r=0.071)	*p=0.047 (r=0.374)
k opioid	-	-	-	p=0.787 (r=-0.056)	p=0.228 (r=0.240)
Ki-67	-	-	-	-	p=0.575 (r=0.120)
TUNEL	-	-	-	-	-

*p<0.05, Spearamn’s correlation. In tumor, k opioid was directly correlated with µ opioid (p = 0.001) and TUNEL (p = 0.028), however ki67 was inversely correlated with VEGF (p = 0.018). In control tissue µ opioid and VEGF were directly correlated (p = 0.044) and in Lymph node metastasis k opioid was directly correlated with µ opioid (p = 0.001) and inversely correlated with ki-67. Additionaly, in lymph node metastasis TUNEL and VEGF were directly correlated (p = 0.047).

## Discussion

The prevalence and susceptibility of the onset of breast cancer are predominantly higher in Caucasian women, as previously demonstrated (Barros et al., 2013); nevertheless, late diagnosis is more common in African-American populations. In the healthcare field, African-American women are at a disadvantage in terms of higher morbidity and access to treatment (Guimarães and Anjos, 2012). Our study population was comprised primarily of mixed-race patients (n=31/79.5%), who were representative of the epidemiological profile of Brazil. The remarkable diversity of the population has made it difficult to perform race analyses, as multiracial people constitute a majority of population (Gervásio et al., 2001; IBGE, 2020) in the country. Patients ≥60 years of age (n=20/51.3%) were observed, corroborating with the data presented by INCA (2020), which describes breast cancer as being relatively rare before the age of 35 years, with its incidence progressively increasing after the age of 50.

Another demographic aspect reported in the literature, which was also found in the population of this study, is the higher incidence of breast cancer in married women and/or women in relationships, with a higher number of individuals over the age 45 years who were originally from the countryside of the state of Ceará and who had not completed their primary education (Lotti et al, 2008). Some studies have shown notable association between delays in treatment initiation and patients with low educational qualification (Sharma et al., 2012), low family income (Yau et al., 2010), and who are distant from the health-care facility (Ukwenya et al., 2008). The results from these studies show that lower educational levels tended to correlate with older age patients (Guimarães and Anjos, 2012). Nevertheless, a low percentage of patients with a higher educational qualification was also detected. It must be considered that education represents an important factor in the planning and implementation of awareness and prevention of cancer for this population (Guimarães and Anjos, 2012).

The most frequent treatment protocol that was adopted by publicly insured patients was surgery combined with RT and CT, which is directly associated with the tumor size, and all patients were in the T4 stage. Additionally, most patients were alive and had no clinical recurrence until the time of specimen collection, demonstrating that the choice of treatment was adequate.

The immunohistochemical evaluation of the mammary gland biopsies focusing on hormonal receptors revealed that patients presented positivity for ER, PR, and c-erbB2/HER2. These results indicate that ER and PR are highly associated with patient age at diagnosis, being significantly positive in tumors of postmenopausal women. Moreover, most PR-positive cases were also ER-positive, and this finding was similar to that reported by Brito (2007). In a study by Gupta et al., (2015), the authors found that about two thirds of breast carcinomas are ER and/or PR-positive, and this positivity has an inverse relationship with tumor size and histological grade. PR positive tumors have been shown to have good prognosis and better responses to hormone therapy. Patients with RP positive tumors were reported to have a longer disease-free time and longer survival rate (Brito et al., 2007). In our study, it was shown that 51.4% of the cases were positive for Ki-67 in 25% of the image fields analyzed, which is consistent with previous studies (Quintão et al., 2016).

Positivity for Ki-67 protein is an indicator of poor prognosis and its overexpression correlates with high mitotic activity, cellular undifferentiation, and increased tendency toward invasion (Marinho et al., 2008). Despite not being statistically significant, a higher percentage of immunostaining was observed in the tumor parenchyma of patients who used opioids during the clinical course of the disease.

The TUNEL assay results demonstrated that patients with the luminal A molecular subtype exhibit a significant increase in tumor cell staining when compared to patients with the luminal B subtype. Our data is substantiated by those from Barros et al., (2015), which suggests that the luminal B variant presents a high apoptosis index (Barros et al., 2013). Recent studies have also shown that the luminal A subtype usually exhibits a Ki-67 index below 14% of malignant neoplastic cells, resulting in a better prognosis (Barros et al., 2013).

In contrast, luminal subtype B tumors have a predominantly proliferative index, which results in a worse prognosis compared to luminal A tumors (Barros et al., 2013). Luminal subtype B was significantly associated with a higher risk of recurrence and lower disease-free survival compared with Luminal subtype A in all categories of systemic adjuvant treatment (Cirqueira et al., 2011).

Regarding the association between recurrence and immunoexpression of opioid receptors, patients with recurrence showed a decrease in the expression of κ-opioid receptors in the metastatic lymph nodes. Previous studies with other types of tumors have shown that the low expression of κ-opioid receptors in metastatic lymph nodes might be associated with better prognosis, resulting in significant improvement in patient survival (Zhang et al., 2013).

In this study, high VEGF scores were shown in both luminal A and luminal B phenotypes. There were no differences between these subtypes in tumor, peritumoral area, or lymph node metastasis. While there were also no differences between recurrent and non-recurrent tumors, recurrence was found to be inversely associated with VEGF immunostaining. However, Ki-67 was inversely correlated with VEGF in the tumor, in µ-opioid control tissue, and VEGF were directly correlated and, in lymph node metastases, TUNEL and VEGF were directly correlated. Although there is no direct relationship between VEGF and opioid receptors in the tumor and in lymph node metastasis, previous studies have shown that the activation of these receptors in injured tissues causes an increase in VEGF expression, favoring angiogenesis (Singleton and Moss, 2010). There is still a consensus about a pathway related to the increase or decrease in VEGF and opioid activation. However, it is believed that the activation of µ-opioid receptors suppresses tumor angiogenesis through the inhibition of hypoxia-inducible transcription factors (HIFs), which increases the expression of VEGF (Kohei Yamamizu, 2014; Lunger et al., 2016 ). \

In our study, an inverse relationship was observed between activation of the receptor µ-opioid and Ki-67 in nodal metastasis, in addition to a direct association with TUNEL in the primary tumor. Previous studies have associated the chronic use of morphine with inhibition of tumor growth (Yeager and Colacchio, 1991; Tegeder et al., 2003; Koodie et al., 2010, 2014), which includes pathways involving the activation of p53 (Tegeder et al., 2003). Conversely, experimental studies and retrospective clinical analyses that involve opioids have shown the promotion of cancer progression and patient survival through different mechanisms (Zagon et al., 1983; Gupta et al, 2002).

A previous study (Sjögren et al., 2016) demonstrated that patients with advanced cancer and intractable pain who received intrathecal morphine had a longer survival than those who received systemic morphine exclusively. Other studies have demonstrated that κ agonists decrease tumor growth in a dose-dependent manner by inhibiting angiogenesis (Mathew et al, 2012), which highlights the importance of these receptors and their effect on cell growth.

In conclusion, we noted that there was no breast cancer, and immunoexpression of µ- and κ-opioid receptors correlated directly in the studied cases. Although κ-opioid shows less immunoexpression in nodal metastasis of tumors with recurrence, compared to tumors without recurrence, no relationship was observed between this marker and Ki-67 decrease. However, in a primary tumor, a direct relationship between µ-opioid and markers of cell death was observed. In nodal metastasis, we show that this same marker was indirectly related to the cell proliferation marker. Neither of the two receptors was expressed in either the primary tumor or nodal metastasis in relation to VEGF.

Future prospective studies are needed to functionally validate these markers and investigate the consequences of opioid use at diagnosis and throughout the course of the disease, as well as in the different molecular subtypes of breast cancer.
